# Association between human leukocyte antigen (HLA) and end-stage renal disease (ESRD): a meta-analysis

**DOI:** 10.7717/peerj.14792

**Published:** 2023-02-13

**Authors:** Naila Noureen, Nousheen Zaidi

**Affiliations:** 1 Cancer Biology Lab, Institute of Microbiology and Molecular Genetics, University of the Punjab, Lahore, Pakistan; 2 Cancer Research Center (CRC), University of the Punjab, Lahore, Punjab, Pakistan

**Keywords:** ESRD, HLA polymorphism, Kidney disease, HLA

## Abstract

**Objectives:**

We recently studied the association between various human leukocyte antigen (HLA) alleles and end-stage renal disease (ESRD). According to our analysis, HLA-B*50 and HLA-DQA1*3 alleles were positively associated with ESRD, while B*40, DRB1*12, DRB1*13, and DQA1*6 alleles were negatively associated with ESRD. However, a single case-control study does not have enough statistical power to evaluate the possible impact of genetic polymorphism on any disease. Hence, the main objective of this meta-analysis is to determine the association between these abovementioned HLA alleles and ESRD.

**Design:**

MEDLINE/PubMed, EMBASE, Web of Science, and Cochrane databases were searched through December 2020 for case-control studies on the associations between HLA polymorphisms and ESRD. Independent reviewers screened the texts of potentially eligible studies and assessed the risk of bias. The meta-analysis was conducted based on the checklists and guidelines based on PRISMA.

**Results:**

We identified 26 case-control studies comprising 1,312 ESRD and 3,842 healthy subjects. A non-significant positive association was observed between HLA-B*50 (OR = 1.02, 95% CI [0.90, 1.24]), HLA-B*40 (OR = 1.75, 95% CI [0.98, 3.2]), HLA-DQA1*3, (OR = 1.17, 95% CI [0.74, 1.84]), DRB1*12 (OR = 1.05, 95% CI [0.94, 1.18]) alleles and ESRD. In addition, a non-significant negative association was observed between HLA-DRB1*13 (OR = 0.90, CI [0.81, 1.01]), HLA-DQB1*6 (OR = 0.79, 95% CI [0.58, 1.07]) alleles and ESRD.

**Conclusions:**

Our meta-analysis indicates no significant association between HLA-B*50, HLA-DQA1*3, B*40, DRB1*12, DRB1*13, and DQA1*6 alleles and ESRD. Further studies with larger sample sizes and adjustments for confounders are required to confirm these conclusions.

## Introduction

End-stage renal disease (ESRD) is the last stage of chronic kidney disease (CKD) (Chiou & Chung, 2012). The pace of ESRD progression varies among individuals, and one of the reasons for these variations could be a different genetic susceptibility to the disease. It has been reported that individuals with a family history of ESRD display a three- to nine-fold greater risk of ESRD (Ferguson, Grim & Opgenorth, 1988; Freedman et al., 1993; Spray et al., 1995). This familial clustering of nephropathy has been observed across various population groups studied. It has been suggested that environmental factors, genes, and their interactions may cause enhanced susceptibility to ESRD (Berger et al., 2003). Numerous studies have searched for genetic factors associated with ESRD risk, and different candidate genes that could make an individual susceptible to ESRD have been identified (Cao et al., 2014).

Human leukocyte antigen (HLA) alleles are known to confer susceptibility to various kidney diseases (Robson et al., 2018). Multiple studies have investigated the possible associations between different HLA types and ESRD (Ademović-Sazdanić & Vojvodić, 2019; Bhallil et al., 2017; Cao et al., 2014; Dai et al., 2015a; Hamdi, Al-Hababi & Eid, 2017; Mosaad et al., 2014; Rivera et al., 2012; Shao et al., 2018). However, the data from these studies show contradictory and inconsistent evidence. We recently revisited the association between ESRD and HLA antigens, comparing polymorphism at HLA-A, -B, -C, -DRB1, -DQB1, and DQA1 loci in ESRD patients and controls (Noureen et al., 2020). Our study, focused on the Pakistani population, showed that several HLA alleles displayed a significant positive or negative association with ESRD. According to this analysis HLA-B*50 and HLA-DQA1*3 alleles were positively associated with ESRD, indicating that these alleles could confer susceptibility to ESRD. On the other hand, B*40, DRB1*12, DRB1*13, and DQA1*6 were negatively associated with ESRD, indicating their protective role against ESRD. We observed a statistically significant association between these HLA alleles and ESRD. However, a single case-control study does not have enough statistical power to evaluate the possible impact of genetic polymorphism on any medical condition or disease, particularly when the study has a relatively small sample size. Therefore, we aimed to perform a meta-analysis to evaluate the relationship between HLA polymorphism and ESRD risk. We only focused on the HLA alleles found to have a significant association with ESRD in our previous clinical study (Noureen et al., 2020). To the best of our knowledge, this is the first meta-analysis on this subject.

## Material and Methods

The review protocol of this study was pre-registered to Prospero (CRD42021243631).

### Search strategy

Several databases (MEDLINE/PubMed, EMBASE, Web of Science, and Cochrane database) were searched through December 2020 for all publications on the association between HLA polymorphism and ESRD. The studies were identified using a combination of the following keywords: (“HLA” OR “human leukocyte antigen”) AND (“polymorphism” OR “alleles” OR “variant” OR “genotype”) AND (’kidney transplantation’ OR ’renal transplantation’ OR ’kidney graft’ OR ’kidney transplant’ OR ’renal transplant’ OR ’end-stage renal disease’ OR ’renal replacement therapy’ OR dialysis OR hemodialysis OR ’peritoneal dialysis’ OR ’hemodialysis patient’ OR ’ESRD’ OR ’end-stage-renal-disease’).

### Selection/study characteristics

The studies were assessed based on original data analyses to determine their eligibility for inclusion and exclusion in this qualitative analysis. The inclusion criteria for the study were: articles published in English; case-control studies focused on the associations between HLA polymorphisms and ESRD; providing sufficient data for estimating an odds ratio (OR) with a 95% confidence interval (CI). The exclusion criteria for the study were: the reviews, comments, editorials, basic science or animal studies; the studies that were not distributed into patient and control groups; focused only on novel alleles; were duplicate studies and lack of detailed information.

### Data extraction

We reviewed each included study independently to extract the data. The extracted data from the eligible studies include the following information: the first author, year of publication, country, study population, population size, number of cases, and controls for the particular HLA alleles.

### Methodological quality assessment

The quality of the included studies was evaluated using the Newcastle-Ottawa Scale (NOS) scale (http://www.ohri.ca/programs/clinical_epidemiology/oxford.asp). The score of each article was calculated based on three items: selection, comparability, and exposure (maximum score = 9 points). The score of eligible studies must be higher than 5 (Table 1).

**Table 1:** Detailed Newcastle-Ottawa Scale of each included study.

**Study**	**Selection**				**Comparability**		**Exposure**			**NOS**
	**a**	**b**	**c**	**d**	**e**	**f**	**g**	**h**	**i**	
Hamdi, Al-Hababi & Eid (2014)	*	*	*	*	*		*	*		7
Pan et al. (2019)	*	*	*	*	*		*	*		7
Nassar et al. (2017)	*	*	*	*	*		*			6
Mosaad et al. (2014)	*	*	*	*	*	*	*	*		8
Rivera et al. (2012)	*	*	*	*	*		*	*		7
Al-Taie et al. (2012)	*	*	*	*	*		*	*	*	8
Ademović-Sazdanić & Vojvodić (2019)	*	*	*	*	*		*	*		7
Shao et al. (2018)	*	*	*	*	*		*	*		7
Nassar, Al-Shamahy & Masood (2015)	*	*	*	*	*		*			6
Karahan et al. (2009)	*	*	*	*	*		*	*		7
Noureen et al. (2020)	*	*	*	*	*		*	*		8
Chowdhry, Makroo & Kumar (2016)	*	*	*	*	*		*	*		7
Patel et al. (2013)	*	*	*	*	*		*	*		7
Shang et al. (2016)	*	*	*	*	*		*	*		7
Almogren, Shakoor & Hamam (2012)	*	*	*	*	*		*	*		7
Cao et al. (2014)	*	*	*	*	*		*	*		7
Dai et al. (2015a) & Dai et al. (2015b)	*	*	*	*	*		*			6
Hieu, Ha & Nghi (2019)	*	*	*	*	*		*	*		7
Nassar, Al-Shamahy & Masood (2015)	*	*	*	*	*		*			6
Kodaz, Akdeniz & Cengiz (2017)	*	*	*	*	*		*	*		7
Pérez-Luque et al. (2000)	*	*	*	*	*		*	*	*	8
Shaheen et al. (2013)	*	*	*	*	*		*	*		7
Bhallil et al. (2017)	*	*	*	*	*		*			6
Fejzić et al. (2017)	*	*	*	*	*		*	*		7
De Holanda et al. (2018)	*	*	*	*	*		*	*		7
Rostami et al. (2013)	*	*	*	*	*		*	*		7

### Statistical analysis

The meta-analysis was conducted based on the checklists and guidelines based on PRISMA. Review manager 5.4 software was used to perform the meta-analysis. Dichotomous data were reported as OR. The pooled ORs with 95% CIs were calculated to determine the strength of the relationship between the HLA and ESRD. The pooled summary was not used in this analysis with the same study design and statistical models. Heterogeneity among the studies was evaluated using I^2^ statistics. A random-effects model was applied to measure the pooled OR when the I^2^ value was greater than 50%. Otherwise, a fixed-effects model was applied. The publication bias was assessed by using a funnel plot for asymmetry.

## Results

### Characteristics and quality of studies included

**Figure 1:**
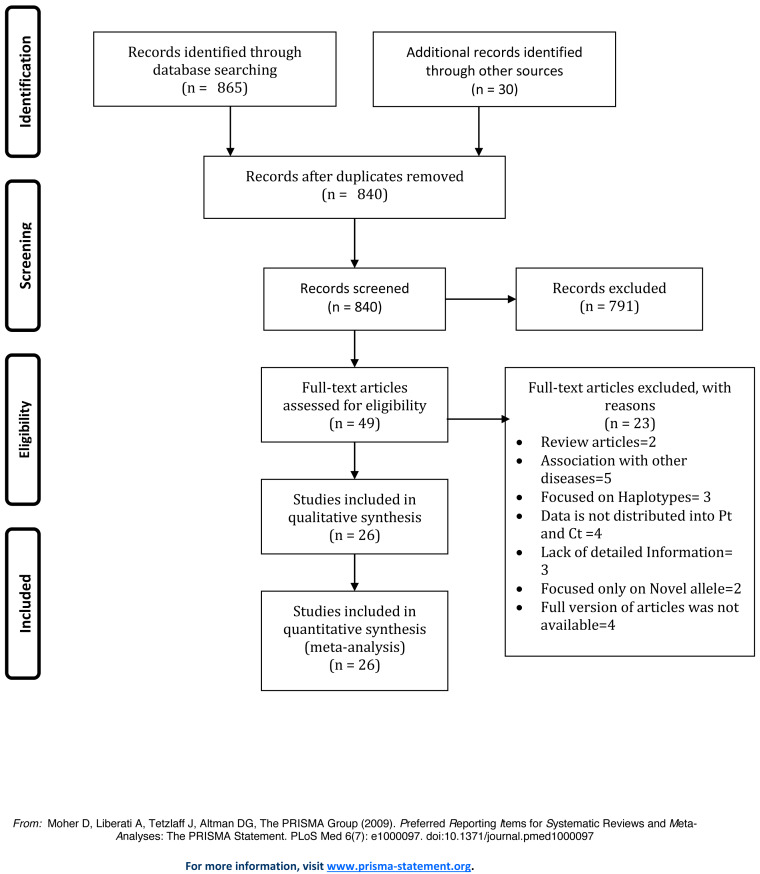
Prisma Flow Diagram of study selection.

We initially obtained 895 articles using the literature retrieval strategy described above. Next, the articles were selected based on the inclusion and exclusion criteria. Figure 1 displays the process of literature selection performed for the presented study. After carefully reviewing the full text of the articles, twenty-six studies were selected for the meta-analysis. Table S1 displays the characteristics of eligible case-control studies included in this meta-analysis.

### Association of HLA B*50 and HLA B*40 with ESRD

**Figure 2:**
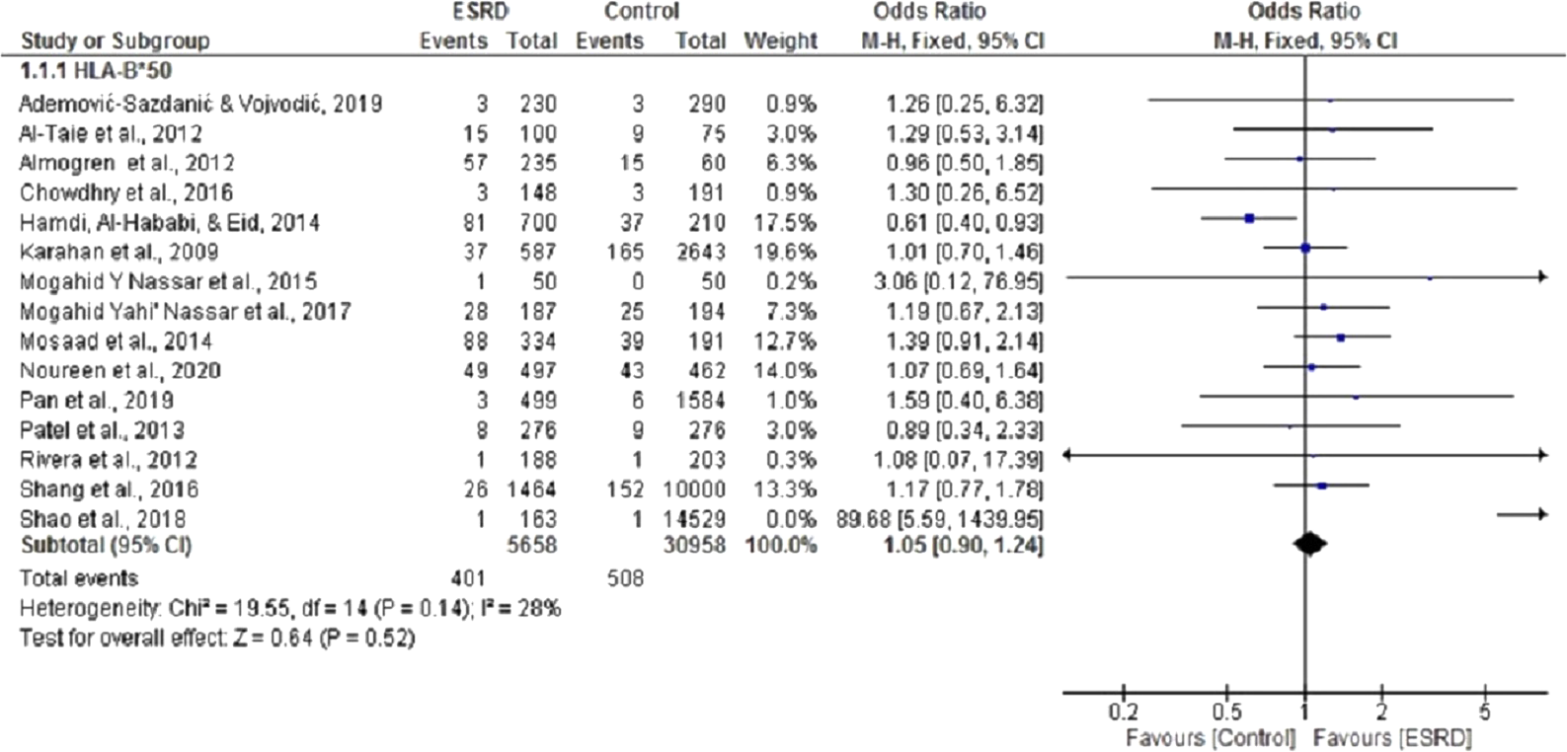
Forest plot of the association of HLA-B*50 with ESRD. Boxes, estimated odd ratios (ORs); bars, 95% confidence intervals (CIs). Diamonds, fixed-effects model 0Rs; width of diamonds; pooled CIs. The size of each box is proportional to the weight of each study in the meta-analysis.

Fifteen (Ademović-Sazdanić & Vojvodić, 2019; Al-Taie et al., 2012; Almogren, Shakoor & Hamam, 2012; Chowdhry, Makroo & Kumar, 2016; Hamdi, Al-Hababi & Eid, 2014; Karahan et al., 2009; Mosaad et al., 2014; Nassar et al., 2017; Nassar, Al-Shamahy & Masood, 2015; Pan et al., 2019; Patel et al., 2013; Rivera et al., 2012; Shang et al., 2016; Shao et al., 2018) case-control studies were selected for meta-analysis to determine the association of HLA B*50 with ESRD. For this analysis, pooled summary OR was 1.02, 95% CI [0.90, 1.24] in a random effect model for ESRD patients compared with healthy individuals. These studies had no significant heterogeneity (Chi^2^ = 19.55, *P* = 0.14, I^2^ = 25%). Three of the selected studies (Almogren, Shakoor & Hamam, 2012; Hamdi, Al-Hababi & Eid, 2014; Patel et al., 2013) showed that HLA B*50 is negatively associated with ESRD, while the rest displayed a positive association. Overall, we did not observe any statistically significant association between HLAB*50 and ESRD (*P* = 0.52) (Fig. 2).

**Figure 3:**
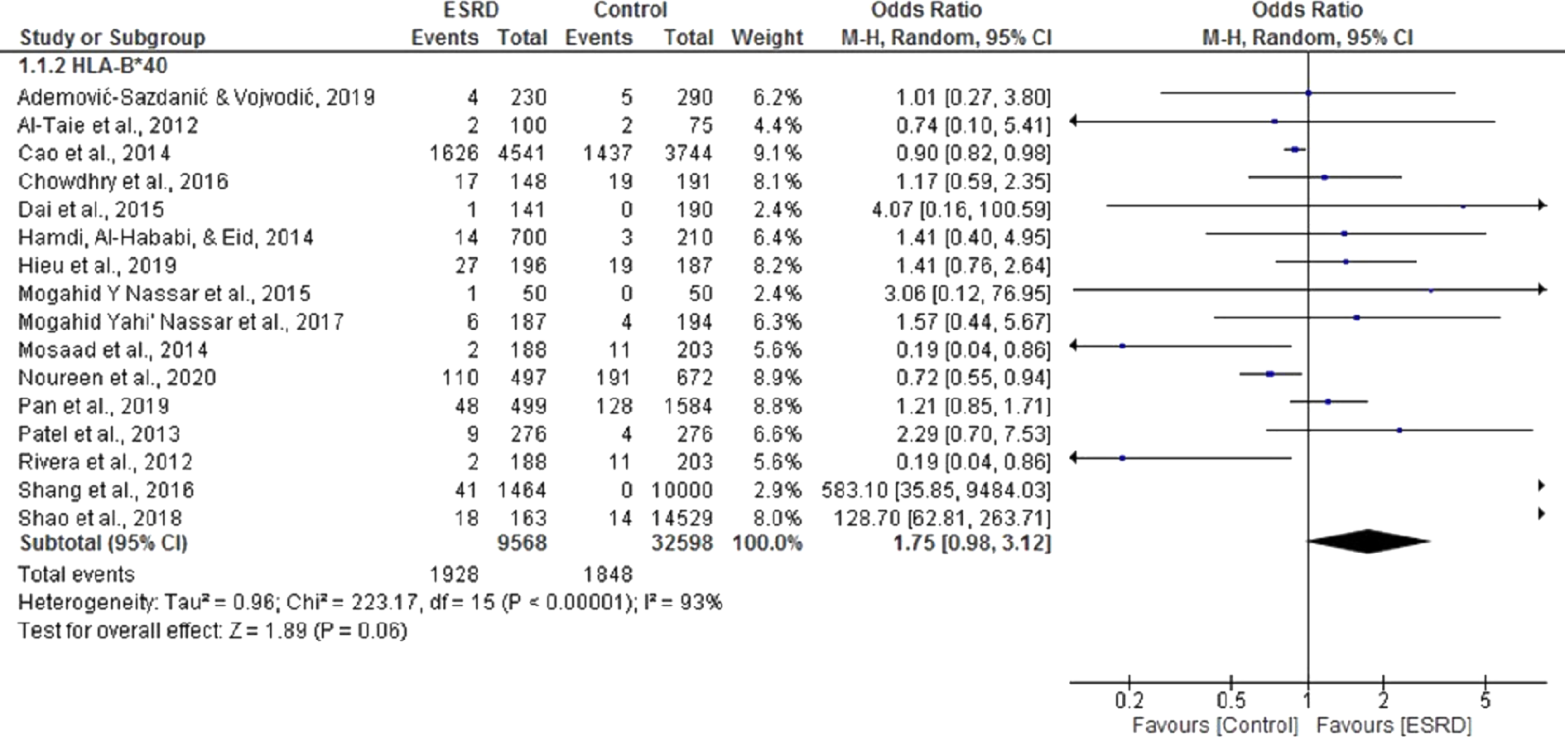
Forest plot of the association of HLA-B*40 with ESRD. Boxes, estimated odd ratios (ORs); bars, 95% confidence intervals (CIs). Diamonds, Random-effects model ORs; width of diamonds; pooled CIs. The size of each box is proportional to the weight of each study in the meta-analysis.

Sixteen (Ademović-Sazdanić & Vojvodić, 2019; Al-Taie et al., 2012; Cao et al., 2014; Chowdhry, Makroo & Kumar, 2016; Dai et al., 2015a; Hamdi, Al-Hababi & Eid, 2014; Hieu, Ha & Nghi, 2019; Mosaad et al., 2014; Nassar et al., 2017; Nassar, Al-Shamahy & Masood, 2015; Pan et al., 2019; Patel et al., 2013; Shang et al., 2016; Shao et al., 2018) case-control studies were included in the meta-analysis to determine the association of HLA B*40 with ESRD. For this meta-analysis, pooled OR was 1.75, 95% CI [0.98, 3.2] in a random effect model for ESRD patients, compared with healthy individuals. There was significant heterogeneity among these studies (Chi^2^ = 223.17, *P* = 0.00001, I^2^ = 93%). Only five of the selected studies showed that B*40 was negatively associated with ESRD (Al-Taie et al., 2012; Cao et al., 2014; Mosaad et al., 2014; Noureen et al., 2020; Rivera et al., 2012). Overall, the results indicated that B*40 was positively associated with ESRD, but the results were not statistically significant (*P* = 0.06) (Fig. 3).

### Association of HLA DRB*12 and DRB*13 with ESRD

**Figure 4:**
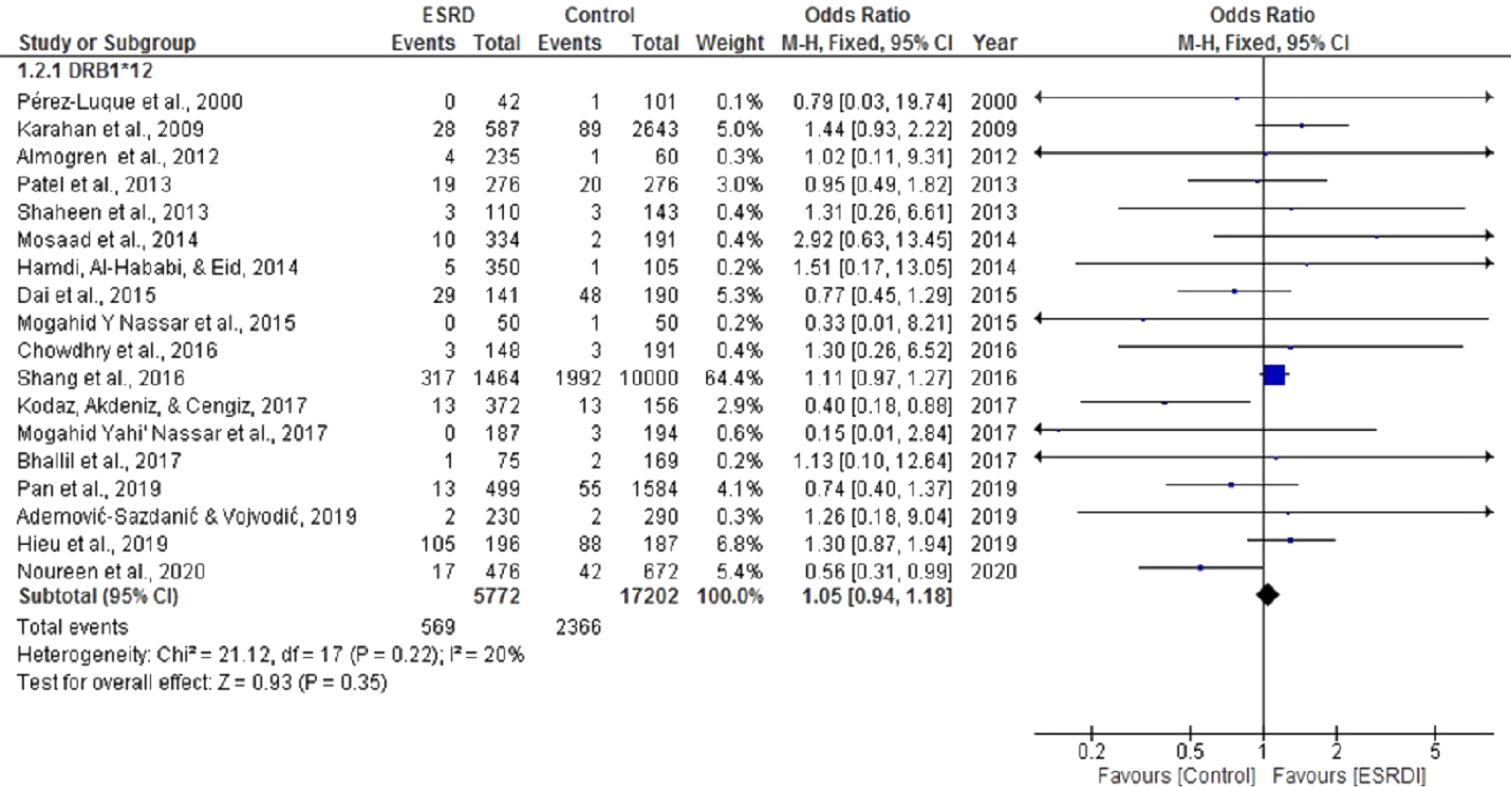
Forest plot of the association of HLA-DRB1*12 with ESRD. Boxes, estimated odd ratios (ORs); bars, 95% confidence intervals (CIs). Diamonds, fixed-effects model 0Rs; width of diamonds; pooled CIs. The size of each box is proportional to the weight of each study in the meta-analysis.

For the meta-analysis aimed to determine the association of HLA DRB*12 with ESRD, eighteen studies were selected (Ademović-Sazdanić & Vojvodić, 2019; Almogren, Shakoor & Hamam, 2012; Bhallil et al., 2017; Chowdhry, Makroo & Kumar, 2016; Dai et al., 2015a; Hamdi, Al-Hababi & Eid, 2014; Hieu, Ha & Nghi, 2019; Karahan et al., 2009; Kodaz, Akdeniz & Cengiz, 2017; Mosaad et al., 2014; Nassar et al., 2017; Nassar, Al-Shamahy & Masood, 2015; Pan et al., 2019; Patel et al., 2013; Pérez-Luque et al., 2000; Shaheen et al., 2013; Shang et al., 2016). These analyses showed a cumulative OR of 1.05, 95% CI [0.94, 1.18] in a random effect model for ESRD patients compared with healthy individuals (Fig. 4). There was no significant heterogeneity among these studies (Chi^2^ = 21.12, *P* = 0.22, I^2^ = 20%). Most of the studies included in this analysis displayed a positive association between DRB*12 and ESRD. Our previously published study (Noureen et al., 2020) and Dai et al. (2015a) also noted a positive correlation between DRB*12 and ESRD (OR = 0.56 CI [0.39,0.99]), (OR = 0.74 CI [0.45,1.29]) respectively. However, the overall pooled estimate of effect was statistically insignificant (*P* = 0.43).

**Figure 5:**
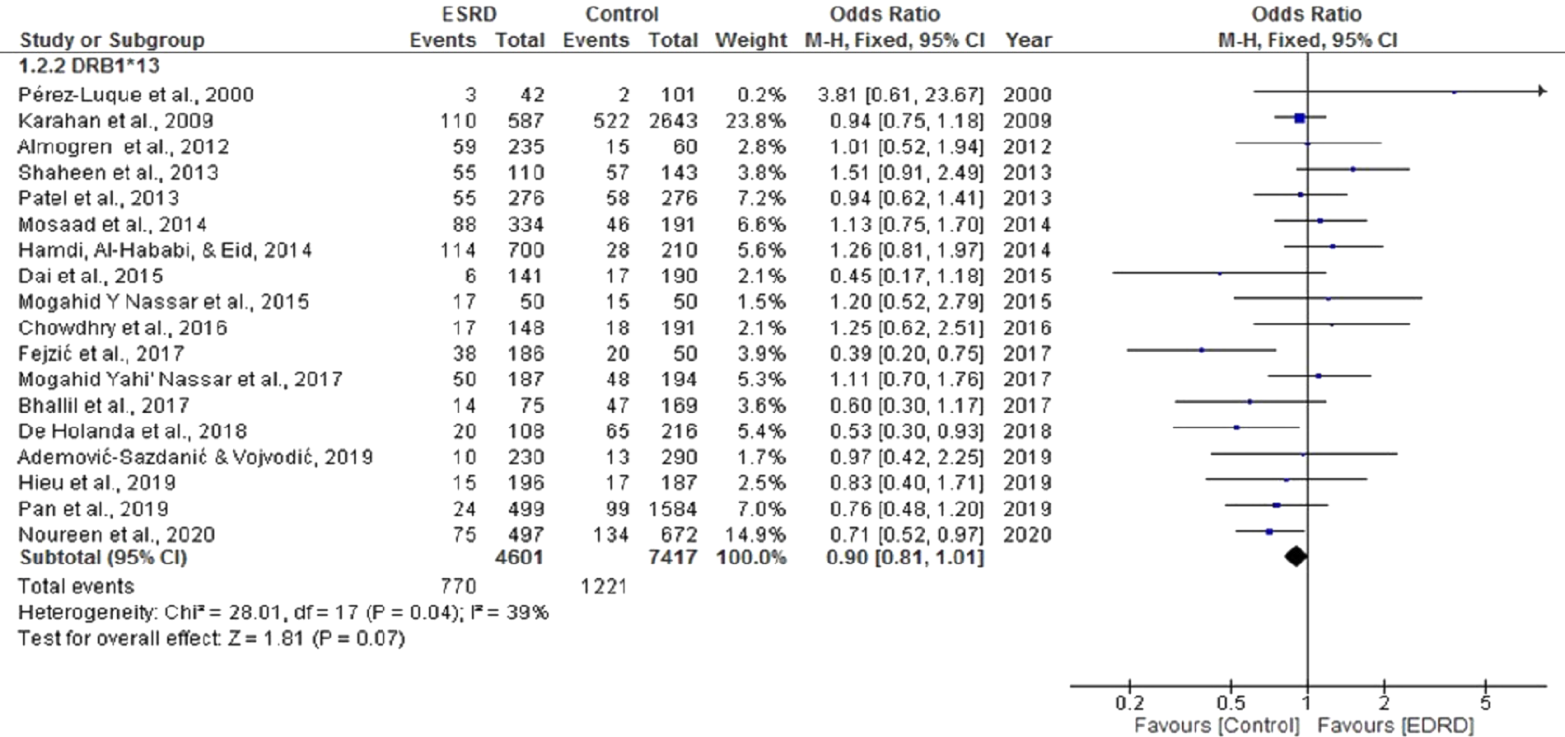
Forest plot of the association of HLA-DRB1*13 with ESRD. Boxes, estimated odd ratios (ORs); bars, 95% confidence intervals (CIs). Diamonds, fixed-effects model 0Rs; width of diamonds; pooled CIs. The size of each box is proportional to the weight of each study in the meta-analysis.

The pooled OR for the meta-analysis aimed to determine the association of HLA DRB*13 with ESRD was 0.90 CI [0.81, 1.01]. Significant heterogeneity was found among the studies (Chi^2^ = 28.01, *P* = 0.04, I^2^ = 39%). Seven studies showed a positive association between HLA DRB*13 and ESRD (Chowdhry, Makroo & Kumar, 2016; Hamdi, Al-Hababi & Eid, 2014; Mosaad et al., 2014; Nassar et al., 2017; Nassar, Al-Shamahy & Masood, 2015; Pérez-Luque et al., 2000; Shaheen et al., 2013). However, there was no statistically significant negative association between ESRD and DRB*13 (OR = 0.90, CI [0.81, 1.01]) (*P* = 0.07) (Fig. 5).

### Association of HLA DQB1*6 and DQA1*3 with ESRD

**Figure 6:**
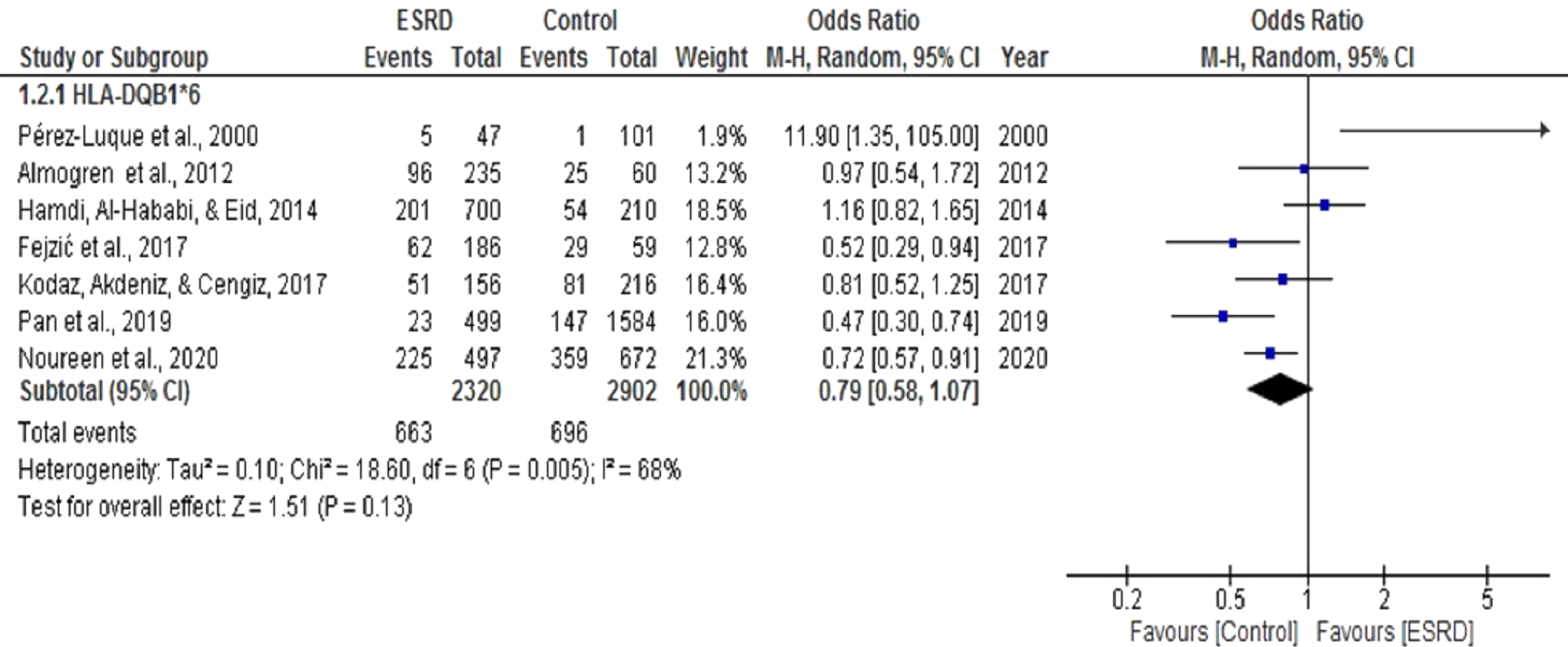
Forest plot of the association of HLA-DQB1*6 with ESRD. Boxes, estimated odd ratios (ORs); bars, 95% confidence intervals (CIs). Diamonds, Random-effects model ORs; width of diamonds; pooled CIs. The size of each box is proportional to the weight of each study in the meta-analysis.

Seven (Almogren, Shakoor & Hamam, 2012; Fejzić et al., 2017; Hamdi, Al-Hababi & Eid, 2014; Kodaz, Akdeniz & Cengiz, 2017; Noureen et al., 2020; Pan et al., 2019; Pérez-Luque et al., 2000) case-control studies were selected for meta-analysis to determine the association of HLA DQB1*6 with ESRD. The pooled OR summary in a random effect model for ESRD patients, compared with healthy individuals for this analysis. The heterogeneity among these studies (Chi^2^ = 18.60, *P* = 0.005, I^2^ = 68%) was statistically significant (Fig. 6). Overall, the meta-analysis results show that DQB1*6 was negatively associated with ESRD, indicating its protective role towards ESRD. There was only one study by Hamdi, Al-Hababi & Eid (2014) that showed a positive association of DQB1*6 with ESRD (OR = 1.16, CI [0.82, 1.65]). However, the results were statistically non-significant (*P* = 0.13).

**Figure 7:**
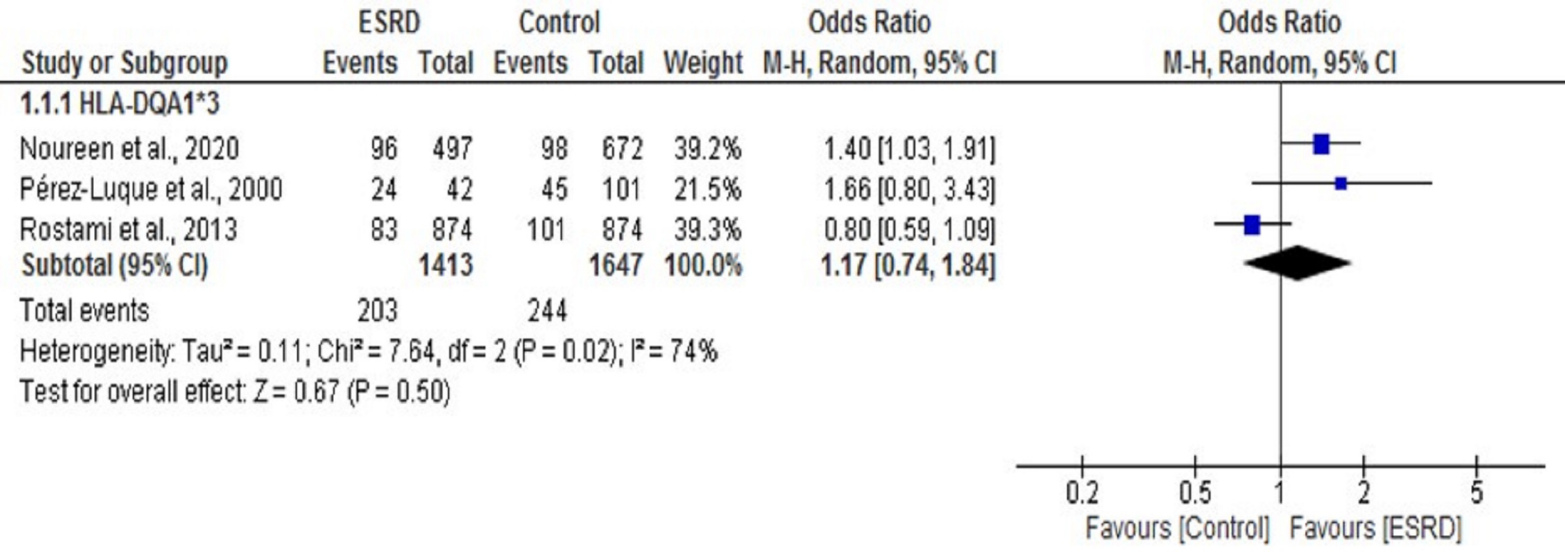
Forest plot of the association of HLA-DQA1*3 with ESRD. Boxes, estimated odd ratios (ORs); bars, 95% confidence intervals (CIs). Diamonds, Random-effects model ORs; width of diamonds; pooled CIs. The size of each box is proportional to the weight of each study in the meta-analysis.

We found only three studies that reported an association between the HLA DQA1*3 allele and ESRD (Pérez-Luque et al., 2000; Rostami et al., 2013). We performed a meta-analysis of these three studies. The OR summary for this analysis was 1.17, 95% CI [0.74, 1.84] (Fig. 7). Our work and a previous study by *Perez-Luque et al.* (Noureen et al., 2020; Pérez-Luque et al., 2000) showed a significant positive association of DQA1*3 with ESRD (OR = 1.40 (1.03, 1.91), 1.66 (0.80, 3.43)), respectively. In contrast, the study conducted by Rostami et al. (2013) showed a significant negative association of DQA1*3 with ESRD (OR = 0.80 (0.59, 1.09)). Overall, no statistically significant association was observed between DQA1*3 and ESRD (*P* = 0.50).

## Discussion

The study conducted by Noureen et al. (2020) showed the association between various HLA alleles and ESRD. The results of the study showed that HLA-B*50 and HLA-DQA1*3 alleles were positively associated with ESRD, while B*40, DRB1*12, DRB1*13, and DQA1*6 alleles were negatively associated with ESRD. However, a single case-control study does not have enough statistical power to evaluate the possible impact of genetic polymorphism on any disease. Hence, the main objective of this meta-analysis is to determine the association between the abovementioned HLA alleles and ESRD.

Our meta-analysis indicates no significant association between HLA-B*50, DQA1*3, B*40, DRB1*12, DRB1*13, and DQA1*6 alleles and ESRD. There is a possibility that the association between the selected HLA alleles and ESRD may vary across diverse populations. For instance, HLA-B*50 is shown to be positively associated with ESRD in Pakistani (Noureen et al., 2020), Chinese (Shang et al., 2016), and Kuwaiti (Mosaad et al., 2014) populations. In contrast, studies conducted on Saudi Arabian (Almogren, Shakoor & Hamam, 2012; Hamdi, Al-Hababi & Eid, 2014) and Indian (Patel et al., 2013) populations displayed a negative association. The studies conducted on the Indian population (Chowdhry, Makroo & Kumar, 2016; Patel et al., 2013) showed that HLA-B*40 is positively associated with ESRD. The studies conducted on the Chinese ethnic groups (Shang et al., 2016; Shao et al., 2018) showed that HLA B*40 is positively associated with ESRD, while one of the opposing findings reported by Cao et al. (2014) showed that HLA-B*40 is protective towards ESRD in Chinese population. The Saudi population (Almogren, Shakoor & Hamam, 2012; Hamdi, Al-Hababi & Eid, 2017) showed that DRB1*12 is positively associated with ESRD, while the Chinese and Taiwanese population (Dai et al., 2015b; Pan et al., 2019) indicated that DRB1*12 is protective towards ESRD. The studies conducted on the Yemen population (Nassar et al., 2017; Nassar, Al-Shamahy & Masood, 2015) indicated that DRB1*13 is positively associated with ESRD, while a study on a Pakistani population (Noureen et al., 2020) showed DRB1*13 protective role towards ESRD.

These differences could be driven by interactions between genetic background and environmental factors, including differences in geographic location and lifestyle factors (Jiang et al., 2019). The variations in the frequency of various HLA alleles in different ethnic groups are the basic reason for heterogeneity. The genetic differences among various ethnic groups also play an important role in the heterogeneity of other studies. However, environmental factors also contribute to the development of ESRD in different ethnic groups.

Moreover, it is also crucial to consider that these discrepancies could be due to the diversity in the designs of studies included in this meta-analysis. It is important to note that the size of each study is a useful to measure reliability. Most studies included in the meta-analysis have a relatively small sample size, which is the main cause of variations among different studies. Smaller studies helpful in summarizing the presented information and producing hypotheses for future investigations. While larger studies are more authentic and reliable and can be clinically useful. We should also take the following limitations of this meta-analysis into account: (1) only unadjusted values were used to evaluate the strength of the association, and (2) selection bias, *i.e.,* only the studies written in English were included in the analysis. Further studies with larger sample sizes and adjustments for confounders are required to confirm our conclusions.

##  Supplemental Information

10.7717/peerj.14792/supp-1 Supplemental Information 1 PRISMA checklist

10.7717/peerj.14792/supp-2 Supplemental Information 2 Funnel plot for HLA-B*40 allele

10.7717/peerj.14792/supp-3 Supplemental Information 3 Funnel plot for HLA-B*50 allele

10.7717/peerj.14792/supp-4 Supplemental Information 4 Funnel plot for HLA-DRB1*12 allele

10.7717/peerj.14792/supp-5 Supplemental Information 5 Funnel plot for HLA-DRB1*13 allele

10.7717/peerj.14792/supp-6 Supplemental Information 6 Funnel plot for HLA-DQB1*6 allele

10.7717/peerj.14792/supp-7 Supplemental Information 7 Funnel plot for HLA-DQB1*3 allele

10.7717/peerj.14792/supp-8 Supplemental Information 8 Meta-analysis Rationale

10.7717/peerj.14792/supp-9 Table S1 Characteristics of eligible case-control studies included in this meta-analysis

## Additional Information and Declarations

### Data Availability

The following information was supplied regarding data availability: The PubMed search terms used: (“HLA” OR “human leukocyte antigen”) AND (“polymorphism” OR “alleles” OR “variant” OR “genotype”) AND (‘kidney transplantation’ OR ‘renal transplantation’ OR ‘kidney graft’ OR ‘kidney transplant’ OR ‘renal transplant’ OR ‘end stage renal disease’ OR ‘renal replacement therapy’ OR dialysis OR hemodialysis OR ‘peritoneal dialysis’ OR ‘hemodialysis patient’ OR ‘ESRD’ OR ‘end-stage-renal-disease’).
