# Supplementary figures and images for: Association between human leukocyte antigen (HLA) and end-stage renal disease (ESRD): a meta-analysis
Source: PeerJ. 2023 Feb 13;11:e14792. doi: 10.7717/peerj.14792 (PMC9933765; doi:10.7717/peerj.14792)

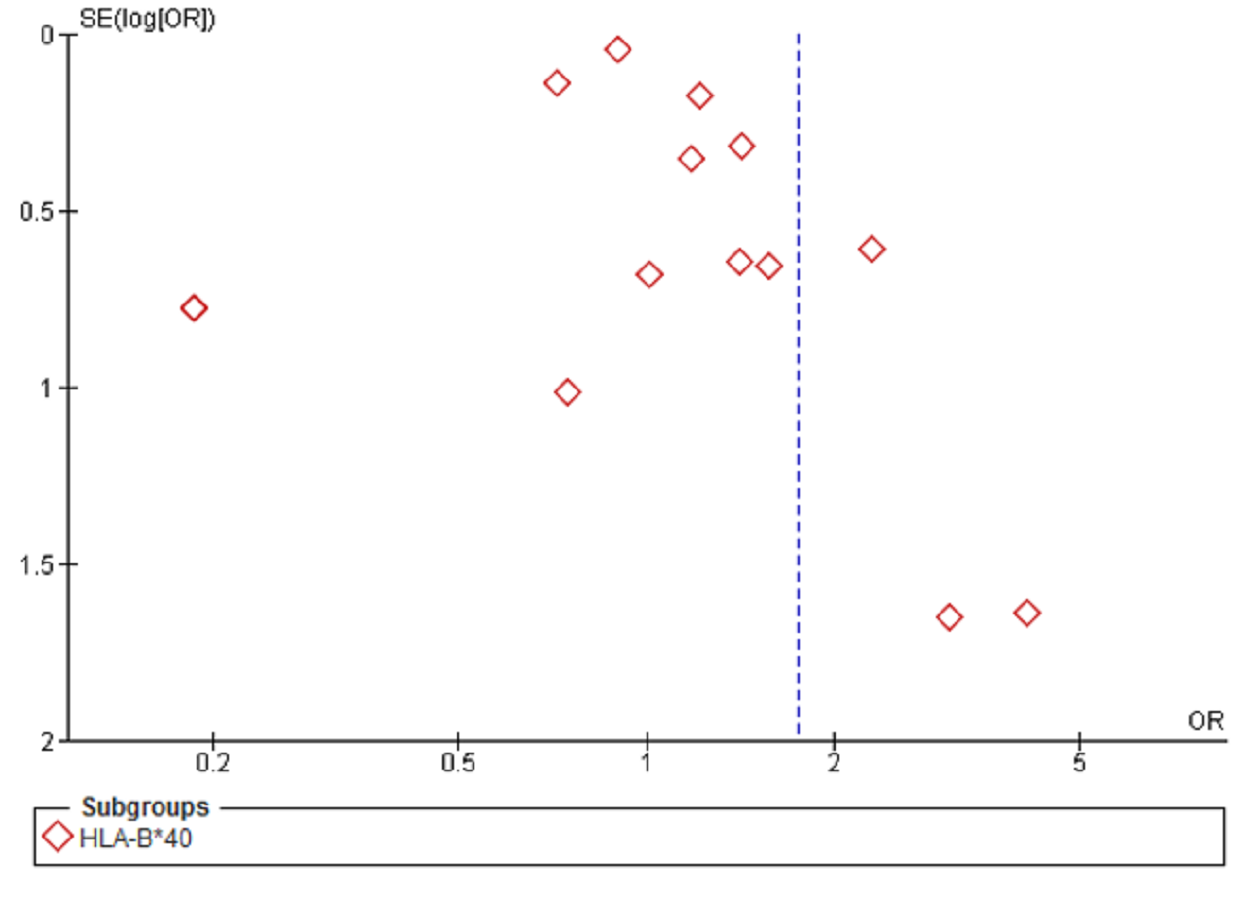

Supplement: Supplemental Information 2 [file peerj-11-14792-s002.png]

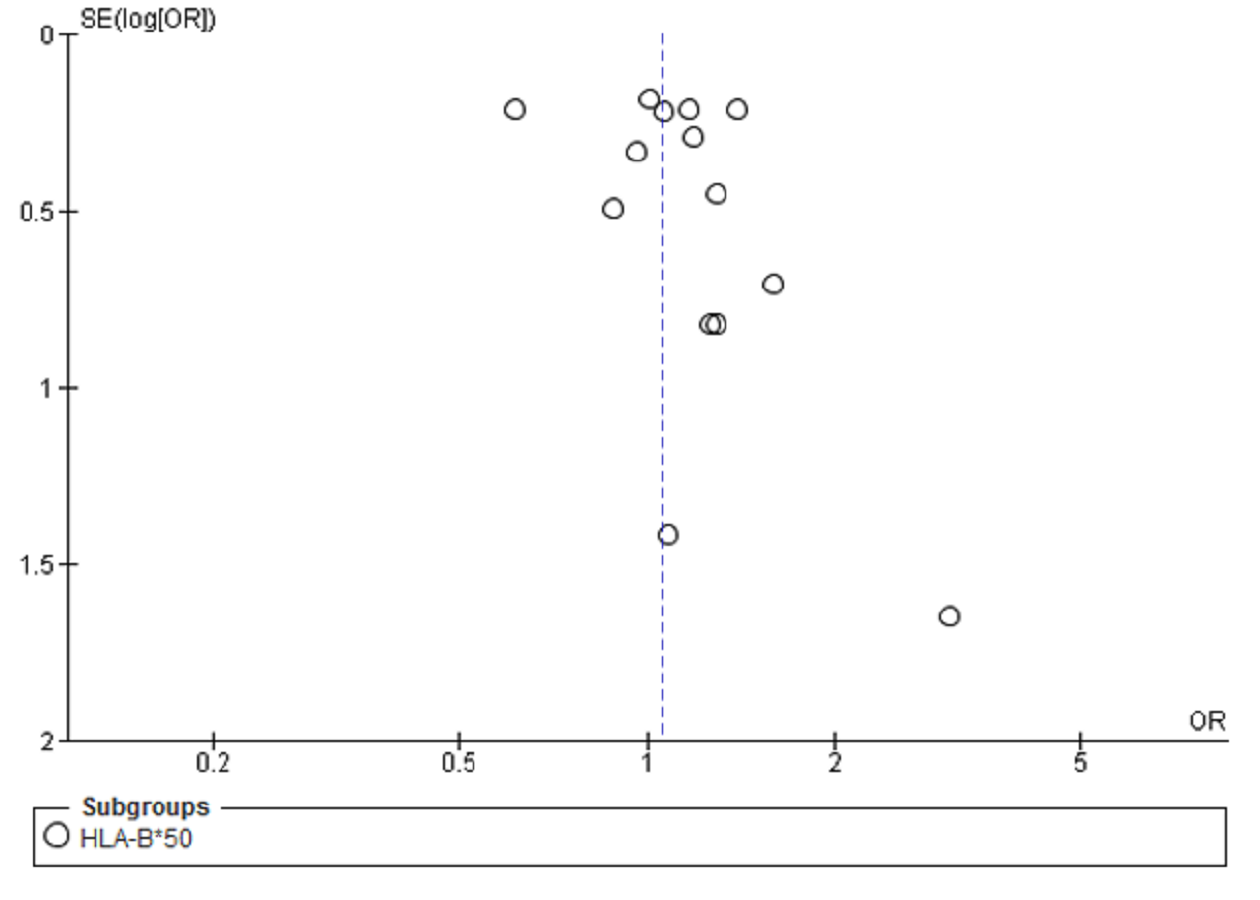

Supplement: Supplemental Information 3 [file peerj-11-14792-s003.png]

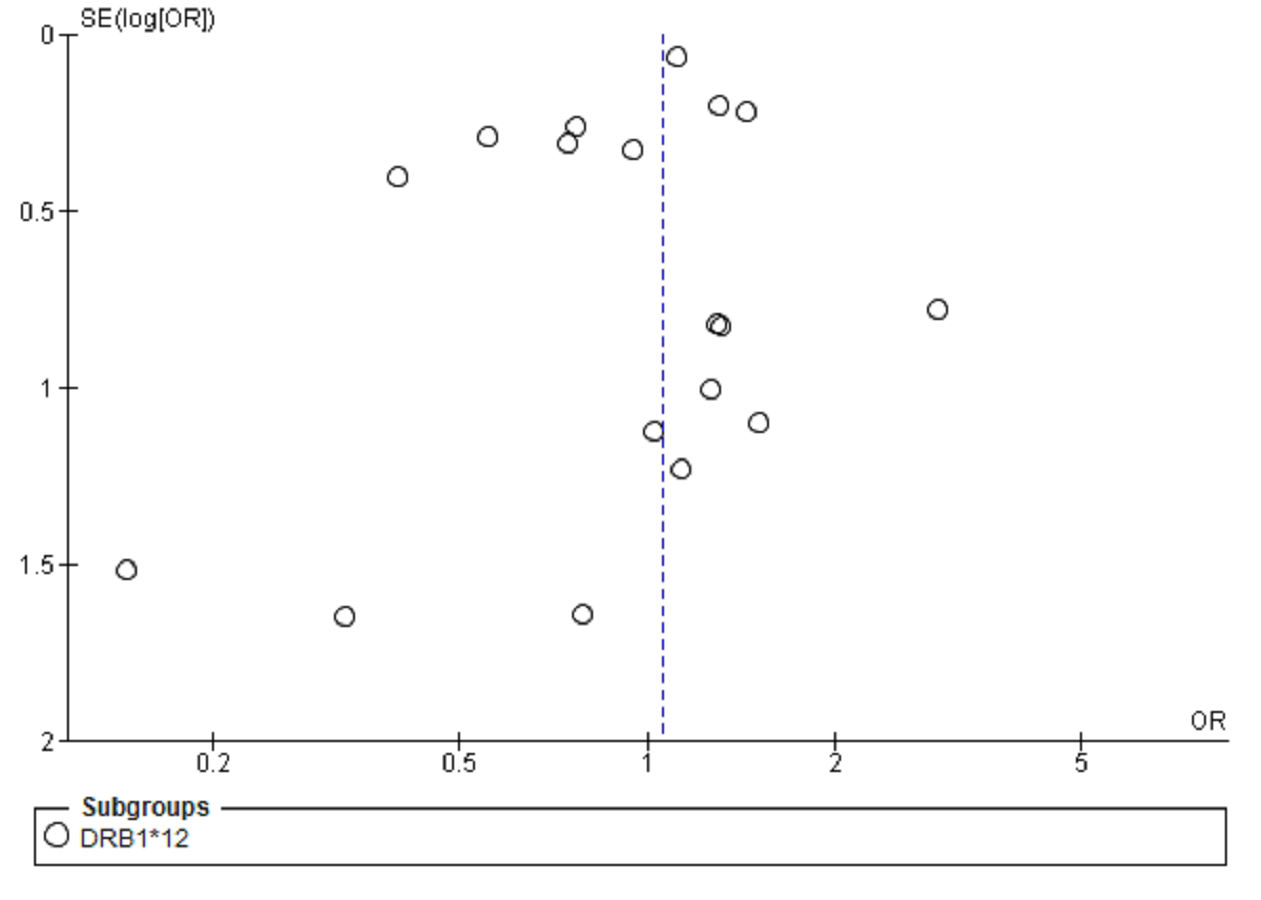

Supplement: Supplemental Information 4 [file peerj-11-14792-s004.png]

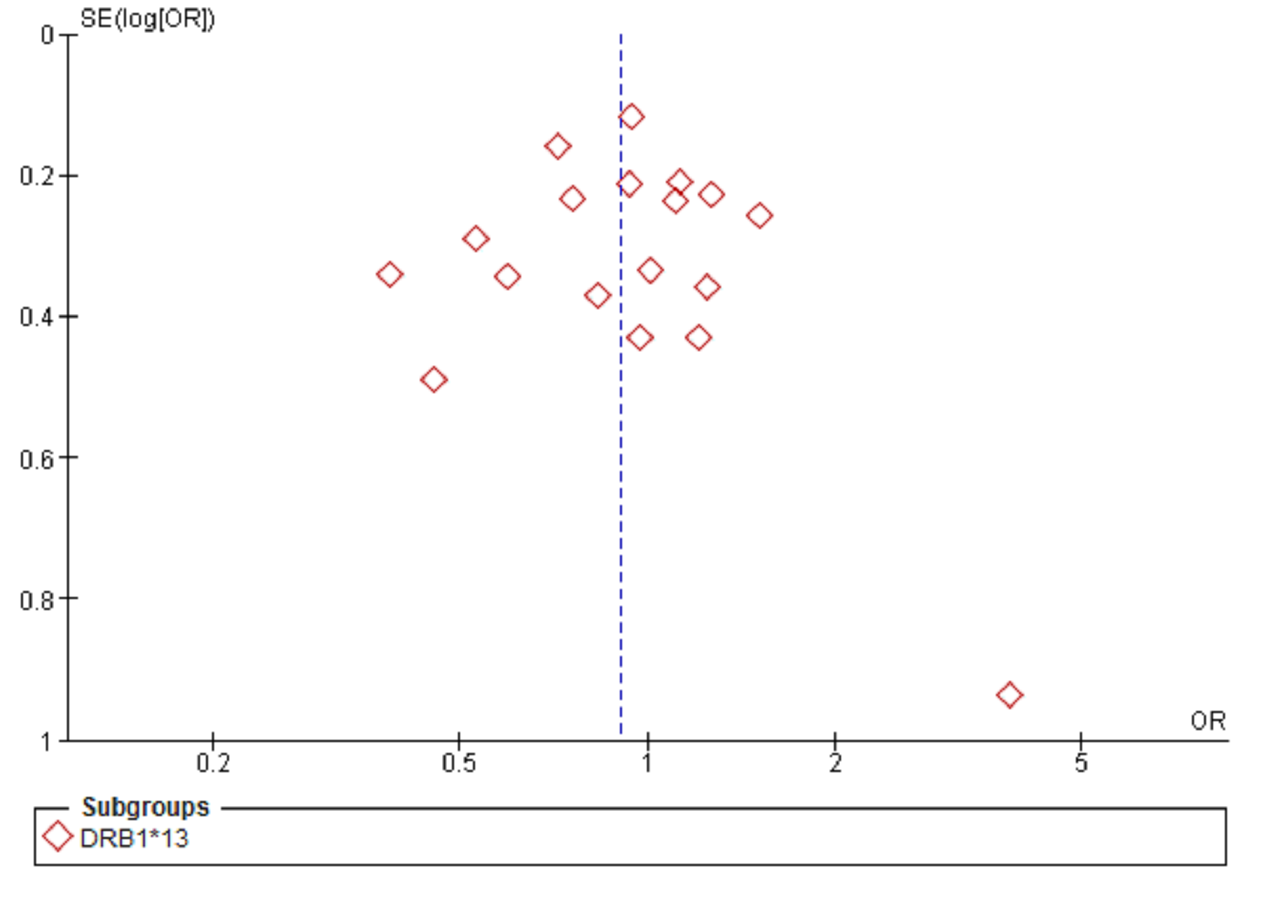

Supplement: Supplemental Information 5 [file peerj-11-14792-s005.png]

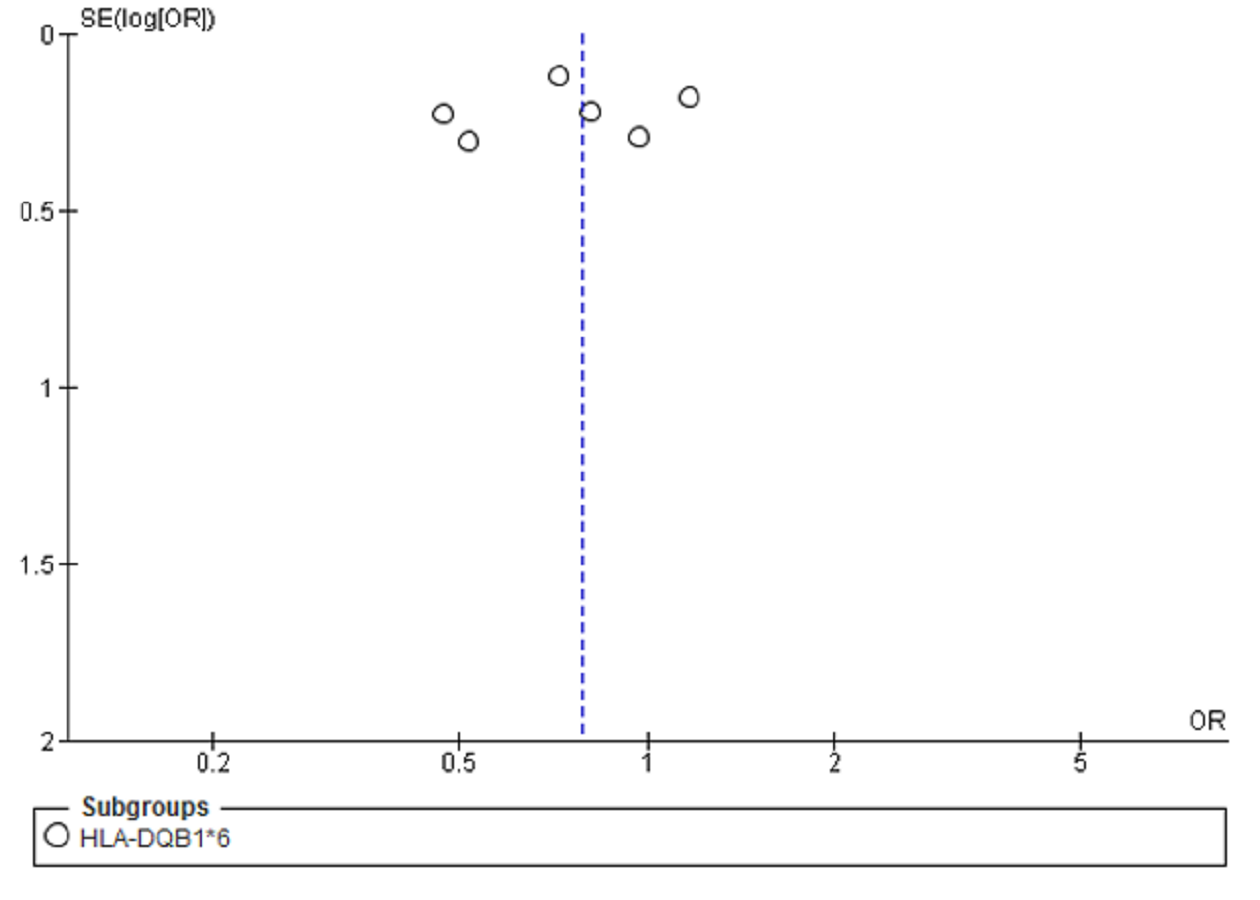

Supplement: Supplemental Information 6 [file peerj-11-14792-s006.png]

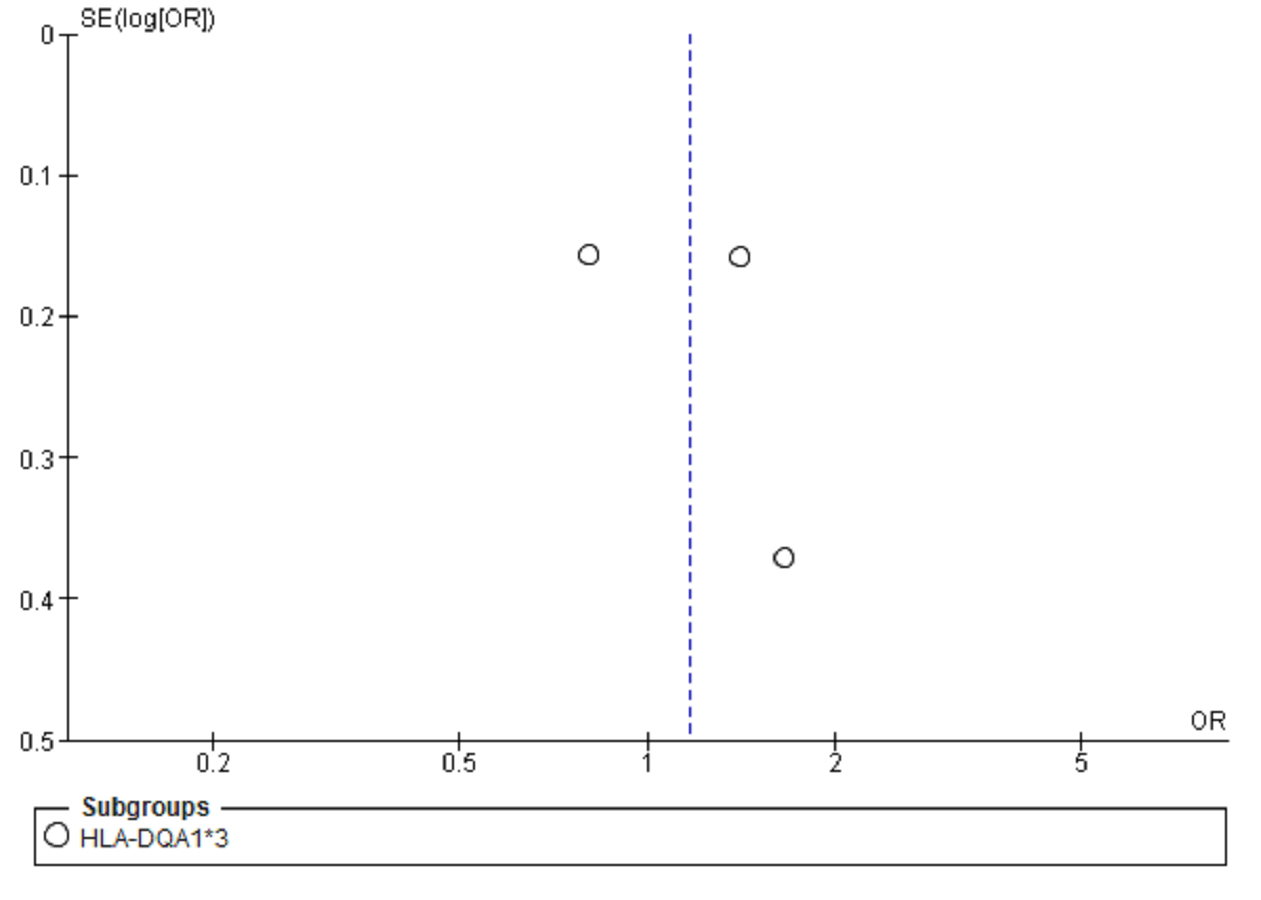

Supplement: Supplemental Information 7 [file peerj-11-14792-s007.png]
