# Supplementary material for: Association between human leukocyte antigen (HLA) and end-stage renal disease (ESRD): a meta-analysis
Source: PeerJ. 2023 Feb 13;11:e14792. doi: 10.7717/peerj.14792 (PMC9933765; doi:10.7717/peerj.14792)
Supplement: Supplemental Information 8 [file peerj-11-14792-s008.docx]

**Systematic Review and/or Meta-Analysis Rationale**

We recently revisited the association between ESRD and HLA antigens, comparing polymorphism at HLA-A, -B, -C, -DRB1, -DQB1, and DQA1 loci in ESRD patients and controls (Noureen et al., 2020). Our study, focused on the Pakistani population, showed that several HLA alleles displayed a significant positive or negative association with ESRD. According to this analysis HLA-B*50 and HLA-DQA1*3 alleles were positively associated with ESRD, indicating that these alleles could confer susceptibility to ESRD. On the other hand, B*40, DRB1*12, DRB1*13, and DQA1*6 were negatively associated with ESRD, indicating their protective role against ESRD. We observed a statistically significant association between these HLA alleles and ESRD. However, a single case-control study does not have enough statistical power to evaluate a possible impact of genetic polymorphism on any medical condition or disease, particularly when the study has a relatively small sample size. Therefore, we aimed to perform a meta-analysis to evaluate the relationship between HLA polymorphism and ESRD risk. We only focused on the HLA alleles found to have a significant association with ESRD in our previous clinical study(Noureen et al., 2020)

Noureen, N., Shah, F. A., Lisec, J., Usman, H., Khalid, M., Munir, R., & Zaidi, N. (2020). Revisiting the association between human leukocyte antigen and end-stage renal disease. *PloS one, 15*(9), e0238878. doi:10.1371/journal.pone.0238878
