# Supplementary material for: Association between human leukocyte antigen (HLA) and end-stage renal disease (ESRD): a meta-analysis
Source: PeerJ. 2023 Feb 13;11:e14792. doi: 10.7717/peerj.14792 (PMC9933765; doi:10.7717/peerj.14792)
Supplement: Table S1 [file peerj-11-14792-s009.docx]

**Table S1:** Characteristics of eligible case-control studies included in this meta-analysis.

| **HLA Alleles** | **Study ID/Year** | **Population size** | **Population type** | **Patient (n)** | **Control (n)** |
| --- | --- | --- | --- | --- | --- |
| **B*50** | ([Hamdi, Al-Hababi et al. 2014](#_ENREF_10)) | ESRD=700  Control= 210 | Saudi Arabia | 81 | 37 |
|  | ([Pan, Ma et al. 2019](#_ENREF_18)) | ESRD=499  Control=1584 | China | 3 | 6 |
|  | ([Nassar, Al-Shamahy et al. 2017](#_ENREF_15)) | ESRD=187  Control=194 | Yemen | 28 | 25 |
|  | ([Mosaad, Mansour et al. 2014](#_ENREF_14)) | ESRD=334  Control=191 | Kuwait | 88 | 39 |
|  | ([Rivera, Márquez et al. 2012](#_ENREF_21)) | ESRD=188  Control=203 | Venezuela | 1 | 1 |
|  | ([Al-Taie, Al-Ghurabi et al. 2012](#_ENREF_2)) | ESRD=100  Control=75 | Iraqi | 15 | 9 |
|  | ([Ademović-Sazdanić and Vojvodić 2019](#_ENREF_1)) | ESRD=230  Control=290 | Serbia | 3 | 3 |
|  | ([Shao, Yang et al. 2018](#_ENREF_26)) | ESRD=163  Control= 14,529 | China | 1 | 1 |
|  | ([Nassar, Al-Shamahy et al. 2015](#_ENREF_16)) | ESRD=50  Control=50 | Yemen | 1 | 0 |
|  | ([Karahan, Seyhun et al. 2009](#_ENREF_12)) | ESRD=587  Control=2643 | Turkish | 37 | 165 |
|  | ([Noureen, Shah et al. 2020](#_ENREF_17)) | ESRD=497  Control=672 | Pakistan | 49 | 43 |
|  | ([Chowdhry, Makroo et al. 2016](#_ENREF_6)) | ESRD= 148  Control= 191 | India | 3 | 3 |
|  | ([Patel, Patel et al. 2013](#_ENREF_19)) | ESRD=276  ESRD=276 | India | 8 | 9 |
|  | ([Shang, Shen et al. 2016](#_ENREF_25)) | ESRD=1464  Control=10,000 | China | 26 | 152 |
|  | ([Almogren, Shakoor et al. 2012](#_ENREF_3)) | ESRD=235  Control=60 | Saudi Arabia | 57 | 15 |
| **B*40** |  |  |  |  |  |
|  | ([Rivera, Márquez et al. 2012](#_ENREF_21)) | ESRD=188  Control=203 | Venezuela | 2 | 11 |
|  | ([Cao, Xie et al. 2014](#_ENREF_5)) | ESRD= 4541  Control= 3744 | China | 1626 | 1437 |
|  | ([Pan, Ma et al. 2019](#_ENREF_18)) | ESRD=499  Control=1584 | China | 48 | 128 |
|  | ([Al-Taie, Al-Ghurabi et al. 2012](#_ENREF_2)) | ESRD=100  Control=75 | Iraqi | 2 | 2 |
|  | ([Hamdi, Al-Hababi et al. 2014](#_ENREF_10)) | ESRD= 700  Control=210 | Saudi Arabia | 14 | 3 |
|  | ([Ademović-Sazdanić and Vojvodić 2019](#_ENREF_1)) | ESRD=230  Control=290 | Serbia | 4 | 5 |
|  | ([Dai, Chu et al. 2015](#_ENREF_7)) | ESRD= 141  Control=190 | Taiwan | 1 | 0 |
|  | ([Hieu, Ha et al. 2019](#_ENREF_11)) | ESRD=196  Control=187 | Vietnam | 27 | 19 |
|  | ([Nassar, Al-Shamahy et al. 2015](#_ENREF_16)) | ESRD=50  Control=50 | Yemen | 1 | 0 |
|  | ([Nassar, Al-Shamahy et al. 2017](#_ENREF_15)) | ESRD=187  Control=194 | Yemen | 6 | 4 |
|  | ([Shao, Yang et al. 2018](#_ENREF_26)) | ESRD=163  Control= 14,529 | China | 18 | 14 |
|  | ([Noureen, Shah et al. 2020](#_ENREF_17)) | ESRD=497  Control=672 | Pakistan | 110 | 191 |
|  | ([Chowdhry, Makroo et al. 2016](#_ENREF_6)) | ESRD=148  Control=191 | India | 17 | 19 |
|  | ([Patel, Patel et al. 2013](#_ENREF_19)) | ESRD=276  Control=276 | India | 9 | 4 |
|  | ([Shang, Shen et al. 2016](#_ENREF_25)) | ESRD=1464  Control=10,000 | China | 41 | 0 |
| **HLA-DRB1** |  |  |  |  |  |
| **DRB1*12** | ([Pan, Ma et al. 2019](#_ENREF_18)) | ESRD=499  Control=1584 | China | 13 | 55 |
|  | ([Kodaz, Akdeniz et al. 2017](#_ENREF_13)) | ESRD= 372  Control=156 | Turkey | 13 | 13 |
|  | ([Mosaad, Mansour et al. 2014](#_ENREF_14)) | ESRD=334  Control=191 | Kuwait | 10 | 2 |
|  | ([Hamdi, Al-Hababi et al. 2014](#_ENREF_10)) | ESRD=350  Control=105 | Saudi Arabia | 5 | 1 |
|  | ([Pérez-Luque, Malacara et al. 2000](#_ENREF_20)) | ESRD=42  Control= 101 | Mexican | 0 | 1 |
|  | ([Ademović-Sazdanić and Vojvodić 2019](#_ENREF_1)) | ESRD=230  Control=290 | Serbia | 2 | 2 |
|  | ([Dai, Chu et al. 2015](#_ENREF_7)) | ESRD=141  Control=190 | Taiwan | 29 | 48 |
|  | ([Hieu, Ha et al. 2019](#_ENREF_11)) | ESRD= 196  Control=187 | Vietnam | 105 | 88 |
|  | ([Nassar, Al-Shamahy et al. 2015](#_ENREF_16)) | ESRD=50  Control=50 | Yemen | 0 | 1 |
|  | ([Nassar, Al-Shamahy et al. 2017](#_ENREF_15)) | ESRD= 187  Control= 194 | Yemen | 0 | 3 |
|  | ([Karahan, Seyhun et al. 2009](#_ENREF_12)) | ESRD= 587  Control=2643 | Turkish | 28 | 89 |
|  | ([Chowdhry, Makroo et al. 2016](#_ENREF_6)) | ESRD=148  Control=191 | India | 3 | 3 |
|  | ([Patel, Patel et al. 2013](#_ENREF_19)) | ESRD=  Control= | India | 19 | 20 |
|  | ([Shaheen, Soliman et al. 2013](#_ENREF_24)) | ESRD=110  Control=143 | Egypt | 3 | 3 |
|  | ([Shang, Shen et al. 2016](#_ENREF_25)) | ESRD=1464  Control=10,000 | China | 317 | 1992 |
|  | ([Bhallil, Ibrahimi et al. 2017](#_ENREF_4)) | ESRD= 75  Control= 169 | Moroccan | 1 | 2 |
|  | ([Almogren, Shakoor et al. 2012](#_ENREF_3)) | ESRD=235  Control=60 | Saudi Arabia | 4 | 1 |
| **DRB1*13** |  |  |  |  |  |
|  | ([Fejzić, Karamehić et al. 2017](#_ENREF_9)) | ESRD=186  Control=50 | Bosnia | 38 | 20 |
|  | ([Pan, Ma et al. 2019](#_ENREF_18)) | ESRD=499  Control=1584 | China | 24 | 99 |
|  | ([Mosaad, Mansour et al. 2014](#_ENREF_14)) | ESRD=334  Control=191 | Kuwait | 88 | 46 |
|  | ([Hamdi, Al-Hababi et al. 2014](#_ENREF_10)) | ESRD=700  Control=210 | Saudi Ariba | 114 | 28 |
|  | ([Pérez-Luque, Malacara et al. 2000](#_ENREF_20)) | ESRD=42  Control=101 | Mexico | 3 | 2 |
|  | ([Ademović-Sazdanić and Vojvodić 2019](#_ENREF_1)) | ESRD=230  Control=290 | Serbia | 10 | 30 |
|  | ([Dai, Chu et al. 2015](#_ENREF_7)) | ESRD=141  Control=190 | Taiwan | 6 | 17 |
|  | ([Hieu, Ha et al. 2019](#_ENREF_11)) | ESRD=196  Control=187 | Vietnam | 15 | 17 |
|  | ([Nassar, Al-Shamahy et al. 2015](#_ENREF_16)) | ESRD=50  Control=50 | Yemen | 17 | 15 |
|  | ([Nassar, Al-Shamahy et al. 2017](#_ENREF_15)) | ESRD=187  Control=194 | Yemen | 50 | 48 |
|  | ([Karahan, Seyhun et al. 2009](#_ENREF_12)) | ESRD= 587  Control=2643 | Turkish | 110 | 522 |
|  | ([Chowdhry, Makroo et al. 2016](#_ENREF_6)) | ESRD=148  Control=191 | India | 17 | 18 |
|  | ([de Holanda, Klumb et al. 2018](#_ENREF_8)) | ESRD=108  Control= 216 | Bosnia | 20 | 65 |
|  | ([Patel, Patel et al. 2013](#_ENREF_19)) | ESRD=276  Control=276 | India | 55 | 58 |
|  | ([Shaheen, Soliman et al. 2013](#_ENREF_23)) | ESRD=110  Control=143 | Egypt | 55 | 57 |
|  | ([Shang, Shen et al. 2016](#_ENREF_25)) | ESRD=1464  Control=10,000 | China | 151 | 1004 |
|  | ([Bhallil, Ibrahimi et al. 2017](#_ENREF_4)) | ESRD=75  CONTROL=169 | Moroccan | 14 | 47 |
|  | ([Almogren, Shakoor et al. 2012](#_ENREF_3)) | ESRD=235  Control=60 | Saudi Arabia | 59 | 15 |
| **HLA-DQB1** |  |  |  |  |  |
| **DQB1*6** | ([Fejzić, Karamehić et al. 2017](#_ENREF_9)) | ESRD=186  Control=59 | Bosnia | 62 | 29 |
|  | ([Pan, Ma et al. 2019](#_ENREF_18)) | ESRD=499  Control 1584 | China | 23 | 147 |
|  | ([Hamdi, Al-Hababi et al. 2014](#_ENREF_10)) | ESRD=700  Control=210 | Saudi Arabia | 201 | 54 |
|  | ([Kodaz, Akdeniz et al. 2017](#_ENREF_13)) | ESRD= 156  Control= 216 | Turkey | 51 | 81 |
|  | ([Pérez-Luque, Malacara et al. 2000](#_ENREF_20)) | ESRD=47  Control=101 | Mexico | 5 | 1 |
|  | ([Almogren, Shakoor et al. 2012](#_ENREF_3)) | ESRD=235  Control=60 | Saudi Arabia | 96 | 25 |
| **HLA-DQA1** |  |  |  |  |  |
| **DQA1*3** | ([Pérez-Luque, Malacara et al. 2000](#_ENREF_20)) | ESRD=42  Control=101 | Mexico | 24 | 45 |
|  | ([Rostami, Shafighiee et al. 2013](#_ENREF_22)) | ESRD=874  Control=874 | Iran | 83 | 101 |
| **DQA1*6** |  |  |  |  |  |

Ademović-Sazdanić, D. and S. Vojvodić (2019). "Human leukocyte antigen polymorphisms as susceptibility risk factors for end stage renal disease." Genetika **51**(2): 607-617.

Al-Taie, L. H., B. H. Al-Ghurabi, et al. (2012). "Frequency of HLA-A and B antigens in Iraqi patients with end-stage renal disease preparing for transplantation." Iraqi Academic Scientific Journal **11**(supplement): 642-648.

Almogren, A., Z. Shakoor, et al. (2012). "Human leucocyte antigens: their association with end-stage renal disease in Saudi patients awaiting transplantation." British journal of biomedical science **69**(4): 159-163.

Bhallil, O., A. Ibrahimi, et al. (2017). "HLA class II with lupus nephritis in moroccan patients." Immunological investigations **46**(1): 1-9.

Cao, Q., D. Xie, et al. (2014). "HLA polymorphism and susceptibility to end-stage renal disease in Cantonese patients awaiting kidney transplantation." PloS one **9**(3): e90869.

Chowdhry, M., R. N. Makroo, et al. (2016). "Genetic diversity through human leukocyte antigen typing in end-stage renal disease patients and prospective donors of North India." Indian Journal of Pathology and Microbiology **59**(1): 59.

Dai, C.-S., C.-C. Chu, et al. (2015). "Association between human leucocyte antigen subtypes and risk of end stage renal disease in Taiwanese: a retrospective study." BMC nephrology **16**(1): 177.

de Holanda, M. I., E. Klumb, et al. (2018). "The prevalence of HLA alleles in a lupus nephritis population." Transplant immunology **47**: 37-43.

Fejzić, E., J. Karamehić, et al. (2017). "HLA Genotyping in Patients with End-Stage Renal Disease Waiting For Cadaveric Renal Transplantation in Federation of Bosnia and Herzegovina." Open access Macedonian journal of medical sciences **5**(1): 1.

Hamdi, N. M., F. H. Al-Hababi, et al. (2014). "HLA class I and class II associations with ESRD in Saudi Arabian population." PloS one **9**(11): e111403.

Hieu, H. T., N. T. Ha, et al. (2019). Association of Human Leukocyte Antigen Haplotypes With End-Stage Renal Disease in Vietnamese Patients Prior to First Transplantation. Transplantation proceedings, Elsevier.

Karahan, G. E., Y. Seyhun, et al. (2009). "Impact of HLA on the underlying primary diseases in Turkish patients with end-stage renal disease." Renal failure **31**(1): 44-49.

Kodaz, H., D. Akdeniz, et al. (2017). "Association Between Human Leukocyte Antigens and Chronic Renal Disease." EURASIAN JOURNAL OF MEDICAL INVESTIGATION **1**(1): 1-5.

Mosaad, Y. M., M. Mansour, et al. (2014). "Association between Human Leukocyte Antigens (HLA-A,-B, and-DR) and end-stage renal disease in Kuwaiti patients awaiting transplantation." Renal failure **36**(8): 1317-1321.

Nassar, M. Y., H. A. Al-Shamahy, et al. (2017). "Human Leukocyte Antigen Class I and II Variants in Yemeni Patients with Chronic Renal Failure." Iranian Journal of Immunology **14**(3): 240-249.

Nassar, M. Y., H. A. Al-Shamahy, et al. (2015). "The Association between Human Leukocyte Antigens and Hypertensive End-Stage Renal Failure among Yemeni Patients." Sultan Qaboos University Medical Journal **15**(2): e241.

Noureen, N., F. A. Shah, et al. (2020). "Revisiting the association between human leukocyte antigen and end-stage renal disease." PLoS One **15**(9): e0238878.

Pan, Q., X. Ma, et al. (2019). "A single center study of protective and susceptible HLA alleles and haplotypes with end-stage renal disease in China." Human Immunology **80**(11): 943-947.

Patel, J. S., M. M. Patel, et al. (2013). "Human leukocyte antigen alleles, genotypes and haplotypes frequencies in renal transplant donors and recipients from West Central India." Indian journal of human genetics **19**(2): 219.

Pérez-Luque, E., J. M. Malacara, et al. (2000). "Contribution of HLA class II genes to end stage renal disease in mexican patients with type 2 diabetes mellitus." Human Immunology **61**(10): 1031-1038.

Rivera, S., G. Márquez, et al. (2012). "HLA class I association with progression to end-stage renal disease in patients from Zulia, Venezuela." Inmunologia **31**(2): 37-42.

Rostami, Z., N. Shafighiee, et al. (2013). "Influence of Donors’ and Recipients’ HLA Typing on Renal Function Immediately After Kidney Transplantation." Nephro-urology monthly **5**(5): 988.

Shaheen, N. M., A. R. Soliman, et al. (2013). "HLA DRB1 alleles and hepatitis C virus infection in chronic kidney disease patients." Ren Fail **35**(3): 386-390.

Shaheen, N. M. H., A. R. Soliman, et al. (2013). "HLA DRB1 alleles and hepatitis C virus infection in chronic kidney disease patients." Renal failure **35**(3): 386-390.

Shang, W., Y. Shen, et al. (2016). "Comparison of HLA-A,-B and-DRB1 loci polymorphism between kidney transplants of uremia patients and healthy individuals in Central China." PloS one **11**(10): e0165426.

Shao, L.-N., Y. Yang, et al. (2018). "Association between the polymorphism of HLA and ESRD in Dalian Han population located in north of China." Immunological investigations **47**(2): 212-219.
